# Resveratrol-Loaded Responsive Core–Shell Tecto Dendrimers Potentiate Hepatocellular Carcinoma Therapy

**DOI:** 10.34133/bmr.0428

**Published:** 2026-10-14

**Authors:** Wenchao Liu, Junjie Liu, Zhenze Zheng, Wenqing Man, Xiaoyang Zhang, Xiaoyi Zheng, Yupeng Wang, Yao Shi, Qinghai Lin, Cong Song

**Affiliations:** ^1^ Department of Clinical Laboratory, Shandong Cancer Hospital and Institute, Shandong First Medical University & Shandong Academy of Medical Sciences, Jinan 250117, China.; ^2^ DWI-Leibniz-Institute for Interactive Materials, Institute for Technical and Macromolecular Chemistry, RWTH Aachen University, Aachen 52074, Germany.; ^3^ Medical Science and Technology Innovation Center, Shandong First Medical University & Shandong Academy of Medical Sciences, Jinan 250117, China.; ^4^ Biomedical Sciences College & Shandong Medical Biotechnology Research Center, National Health Commission Key Laboratory of Biotechnology Drugs, Shandong First Medical University & Shandong Academy of Medical Sciences, Jinan 250117, China.

## Abstract

Hepatocellular carcinoma (HCC) exhibits profound metabolic reprogramming, particularly enhanced glycolytic activity, which represents a therapeutic vulnerability. However, the remarkable adaptability of HCC metabolism allows tumor cells to compensate for metabolic disturbances and preserve cellular homeostasis, reducing the effectiveness of metabolism-targeted strategies. Therefore, strategies sustaining metabolic perturbation beyond tumor adaptive capacity are needed. Herein, we developed a pH-responsive zwitterion-modified core–shell tecto dendrimer nanocarrier (CSTD-PS) for resveratrol (Res) delivery to overcome these limitations and potentiate HCC therapy. CSTD-PS was constructed through dynamic boronate ester-mediated assembly and zwitterionic surface modification, enabling Res encapsulation and pH-responsive release. After Res encapsulation, Res@CSTD-PS exhibited enhanced cellular uptake and nanocarrier-associated tumor localization, resulting in superior antitumor efficacy in HCC models compared with free Res. Mechanistically, Res@CSTD-PS suppressed glycolytic regulators, including hexokinase 2, glucose transporter 1, and pyruvate kinase M2, accompanied by enhanced stress responses (glucose-regulated protein 78 and nuclear factor erythroid 2-related factor 2) and p53/caspase-3-associated apoptotic signaling. Res@CSTD-PS also reduced programmed death-ligand 1 expression, suggesting potential attenuation of immune-evasive characteristics. Collectively, this study introduces a strategy enabled by intelligent dendrimer-based nanomedicine, providing a promising approach for enhancing natural compound-based therapy against HCC.

## Introduction

Hepatocellular carcinoma (HCC) remains one of the most challenging malignancies worldwide, characterized by aggressive progression, high mortality, and limited treatment success in advanced stages [1,2]. Despite advances in surgical, locoregional, and systemic therapies, patients with advanced HCC continue to face limited therapeutic benefits and unfavorable outcomes [3–5]. This therapeutic limitation is largely attributed to tumor heterogeneity, the complex tumor ecosystem, dysregulated signaling networks, and the emergence of therapeutic resistance [6–8]. Therefore, elucidating the key molecular events underlying HCC progression and developing safe, effective, and mechanism-driven therapeutic strategies remain critical priorities in HCC treatment.

Among the emerging hallmarks of HCC progression, metabolic reprogramming, particularly aberrant glycolytic activation, has attracted increasing attention as a therapeutic target [9,10]. This enhanced glycolytic phenotype, known as the Warburg effect, supplies tumor cells with abundant adenosine triphosphate and biosynthetic intermediates, thereby supporting sustained proliferation, invasion, and adaptation to hostile microenvironmental conditions [11–13]. Extensive studies have demonstrated that targeting key glycolytic regulators, including the rate-limiting enzymes and transporters glucose transporter 1 (GLUT1), hexokinase 2 (HK2), and pyruvate kinase M2 (PKM2), can effectively suppress HCC growth [9,14,15]. However, the adaptive capacity of tumor metabolism enables cancer cells to counteract metabolic stress and restore intracellular homeostasis, such as enhanced oxidative phosphorylation or autophagic flux, in response to glycolytic inhibition, allowing them to preserve intracellular energy homeostasis and attenuate therapeutic efficacy [16,17]. Thus, a major challenge in metabolic intervention is to overcome this adaptive capacity, drive metabolic stress beyond the tolerable threshold of tumor cells, and convert stress responses into irreversible cell death signals.

As a natural polyphenolic compound with anticancer activity, resveratrol (Res) has demonstrated considerable therapeutic potential in various malignancies [18,19]. Accumulating evidence indicates that Res exerts antitumor activity by regulating cellular stress responses, including oxidative stress, endoplasmic reticulum stress, and apoptosis-related signaling cascades [20,21]. Moreover, Res can influence glycolytic metabolism through the regulation of key glycolytic regulators, including HK2 and PKM2, which contributes to the disruption of glucose metabolism and metabolic homeostasis in tumor cells [22,23]. These properties provide a potential pharmacological basis for inducing sustained metabolic stress and promoting tumor cell death. However, the intrinsic limitations of Res, including limited water solubility, suboptimal bioavailability, rapid metabolic clearance, and insufficient accumulation at tumor sites, severely restrict its therapeutic translation [24,25]. Developing a delivery system to improve Res stability, enhance tumor delivery, and amplify its metabolic regulatory and anticancer effects is of great importance.

Nanomedicine-based drug delivery systems provide important technological platforms for improving the stability, bioavailability, and delivery efficiency of natural compounds [26–28]. Among these platforms, core–shell tecto dendrimers (CSTDs), specially constructed based on polyamidoamine (PAMAM) dendrimers, have been widely explored for drug delivery due to their unique hierarchical architecture, abundant internal cavities, and tunable surface functionalities [29–32]. Compared with conventional high-generation PAMAM dendrimers, CSTDs are assembled through a core–shell strategy that provides enhanced drug-loading capacity and structural modifiability [32–34]. Such structural flexibility also facilitates the incorporation of responsive linkages and surface functionalities. Our group has previously developed a series of CSTDs through host–guest recognition between fifth-generation (G5) PAMAM dendrimers as the core and third-generation (G3) PAMAM dendrimers as the shell, which have been successfully applied in gene delivery [35,36], chemotherapy [37,38], and tumor imaging/theranostics [39–41]. Building on our previously reported CSTD platforms, the current CSTD-PS system further incorporates zwitterionic 1,3-propane sultone (1,3-PS) surface modification into a dynamic boronate ester-mediated CSTD architecture, aiming to integrate pH-responsive drug release with improved biological interfacial properties. This integrated design provides a rational strategy for the efficient delivery of poorly bioavailable natural compounds such as Res and further extends the CSTD platform toward metabolism-targeted HCC therapy.

Herein, we developed a pH-responsive zwitterion-modified CSTD nanocarrier (CSTD-PS) for efficient Res delivery and enhanced HCC therapy (Fig. 1). The responsive CSTD structure was constructed using phenylboronic acid (PBA)-functionalized G5 PAMAM dendrimers (G5-PBA) as the core and galactose-modified G3 PAMAM dendrimers (G3-Gal) as the shell through dynamic boronate ester linkages, followed by 1,3-PS modification to improve stability, biocompatibility, and antifouling performance (Fig. 1A). After Res loading, Res@CSTD-PS exhibited efficient cellular uptake, acid-responsive drug release, and tumor accumulation. Mechanistically, Res@CSTD-PS disrupted metabolic homeostasis by inhibiting glycolysis, inducing stress responses, activating apoptosis, and reducing programmed death-ligand 1 (PD-L1) expression (Fig. 1B). This study provides a promising strategy for improving the therapeutic performance of natural anticancer agents through intelligent dendrimer-based delivery systems and offers new insights into metabolism-targeted therapy for HCC.

**Fig. 1.**
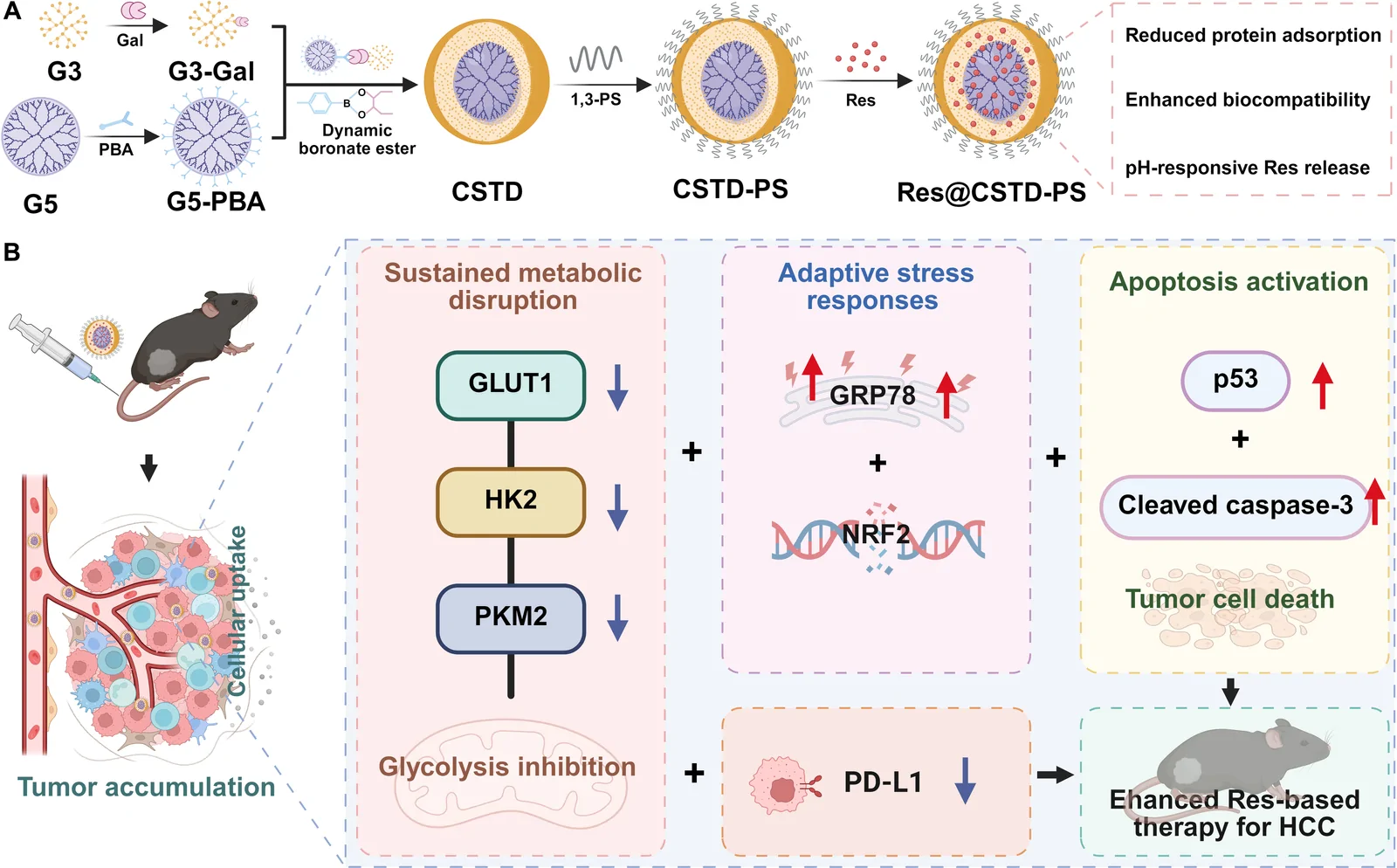
Schematic illustration of the pH-responsive zwitterion-modified core–shell tecto dendrimer nanocarrier (CSTD-PS) for efficient resveratrol (Res) delivery and enhanced hepatocellular carcinoma (HCC) therapy. (A) Construction of the CSTD-PS nanocarrier and formation of Res@CSTD-PS. (B) Proposed mechanism of Res@CSTD-PS-mediated antitumor activity, involving enhanced tumor accumulation, inhibition of glycolytic metabolism, activation of stress responses, induction of apoptotic signaling, and suppression of programmed death-ligand 1 (PD-L1) expression. Created in https://BioRender.com.

## Materials and Methods

### Synthesis of CSTD-PS

CSTD-PS was synthesized through the surface conjugation of 1,3-PS onto CSTD at a molar feed ratio of *n*(CSTD):*n*(1,3-PS) = 1:200. Briefly, 10.0 mg of CSTD was dissolved in 5 ml of water in a reaction flask. Subsequently, 1,3-PS solution (17.3 μl, containing 2.4 mg of 1,3-PS) was added dropwise, and the mixture was stirred at room temperature for 3 d. After completion of the reaction, the mixture was purified by dialysis against ultrapure water using a dialysis membrane with a molecular weight cutoff (MWCO) of 1,000 Da for 3 d. The purified product was lyophilized and further stored at −20 °C.

### Preparation of the Res@CSTD-PS nanocomplex and determination of Res content

Res@CSTD-PS was prepared through hydrophobic-interaction-mediated encapsulation of Res into CSTD-PS. Briefly, 500 μl of the Res methanolic solution (containing 1.12 mg of Res) was added dropwise into the aqueous CSTD-PS (10 mg) solution under continuous stirring overnight in the dark. Subsequently, the mixture was centrifuged at 8,000 rpm for 5 min, and the supernatant containing Res@CSTD-PS was collected. In addition, the precipitate containing unencapsulated Res was collected and dissolved in 1.5 ml of methanol. This solution was analyzed by ultraviolet–visible (UV–vis) spectroscopy at 306 nm to quantify the amount of unencapsulated Res. The standard curve was expressed as *y* = 0.133*x* − 0.0034 (*R*^2^ = 0.9944), where *y* represents the absorbance and *x* represents the Res concentration. The amount of encapsulated Res was calculated by subtracting the amount of unencapsulated Res from the initial amount of Res. The Res loading content (LC) and encapsulation efficiency (EE) were calculated according to the following equations: (a) LC (%) = (mass of encapsulated Res/mass of Res@CSTD-PS) × 100% and (b) EE (%) = (mass of encapsulated Res/initial mass of Res) × 100%.

### In vitro Res release profiles

Res@CSTD-PS (2 mg) was dispersed in 1 ml of phosphate-buffered saline (PBS; pH 7.4, 6.5, and 5.5) and transferred into a dialysis bag (MWCO = 1,000 Da). The dialysis bag was immersed in 14 ml of the corresponding PBS medium and maintained in a shaking incubator at 37 °C. At selected time points, aliquots (1 ml) of the release medium were collected for analysis and immediately replenished with an equivalent volume of fresh PBS to preserve sink conditions. The amount of released Res was quantified by UV–vis spectroscopy, and the cumulative release profile was subsequently determined.

### In vitro evaluations

Mouse HCC cells (Hepa1-6) were cultured in vitro to evaluate the cellular internalization, antitumor activity, and underlying molecular mechanisms of Res@CSTD-PS. The intracellular delivery behavior was investigated using fluorescently labeled Res@CSTD-PS. The therapeutic effects were assessed through cell viability assays, flow cytometric analysis of apoptosis, and 3-dimensional (3D) tumor spheroid models. Furthermore, the regulatory effects of Res@CSTD-PS on glycolytic metabolism, stress-associated responses, apoptotic signaling, and immune-evasive phenotype were investigated by analyzing the expression of glycolysis-related proteins (HK2), stress markers (glucose-regulated protein 78 [GRP78] and nuclear factor erythroid 2-related factor 2 [NRF2]), apoptosis-associated molecules (p53 and cleaved caspase-3), and PD-L1.

### Three-dimensional tumor spheroid assay

Three-dimensional tumor spheroids were established using a 3D cell culture platform according to the standard protocol. Briefly, Hepa1-6 cells were seeded into the 3D culture system and cultured overnight to allow spheroid formation. Subsequently, spheroids were treated with PBS, CSTD-PS, free Res, or Res@CSTD-PS, and bright-field images were collected on days 1, 3, 5, and 7 using an inverted fluorescence microscope. The culture medium was replaced with fresh treatment medium every 2 d. The spheroid size was quantitatively analyzed to evaluate the inhibitory effect of different formulations on tumor spheroid growth.

### Animal experiments

All animal experiments were performed in strict accordance with the requirements of the Animal Care and Use Committee of Shandong First Medical University (approval no. W20251001B009) and Chinese regulations for laboratory animal welfare. A mouse HCC subcutaneous tumor model was established to evaluate the in vivo biodistribution and therapeutic efficacy of Res@CSTD-PS. Briefly, Hepa1-6 cells were subcutaneously injected into the flank region of mice. When tumors reached the desired volume, mice were randomly divided into different groups for subsequent biodistribution and therapeutic efficacy studies.

### In vivo treatment

Tumor-bearing mice were randomly allocated into 4 groups and received intravenous injections of PBS, CSTD-PS, free Res, or Res@CSTD-PS at 3-d intervals. The Res dose was set at 5 mg/kg, and the corresponding amounts of CSTD-PS in the Res@CSTD-PS formulation were used as the carrier dose. Tumor progression was monitored by measuring tumor volume, while body weight was tracked as an indicator of systemic toxicity during treatment. At day 16, mice were sacrificed, and tumors were excised for tumor weight measurement, histological examination, and molecular mechanism analysis.

### In vivo molecular and biochemical assessment

Tumors were collected for histological and molecular analyses at the endpoint of treatment. Therapeutic efficacy was further assessed by terminal deoxynucleotidyl transferase-mediated dUTP nick-end labeling (TUNEL) staining, hematoxylin and eosin (H&E) staining, and Ki67 immunohistochemical staining. In addition, the expression of metabolism-associated proteins (GLUT1, HK2, and PKM2), stress-response proteins (GRP78 and NRF2), apoptosis-associated proteins (p53 and cleaved caspase-3), and PD-L1 was analyzed to clarify the underlying antitumor mechanisms of Res@CSTD-PS.

### Statistical analysis

All experimental data are expressed as mean ± SD from at least 3 independent replicates. Statistical analysis was performed using one-way analysis of variance and Welch’s *t* test where applicable in GraphPad Prism 10.4.1. Statistical significance is denoted as *P < 0.05, **P < 0.01, ***P < 0.001, and ****P < 0.0001.

## Results and Discussion

### Construction and characterization of the responsive CSTD-based nanocarrier

In this work, a pH-responsive zwitterion-modified CSTD-based nanocarrier was constructed through dynamic boronate ester linkages, followed by Res encapsulation to obtain Res@CSTD-PS. The successful synthesis of G3-Gal and G5-PBA, as well as the subsequent formation of CSTD, was first verified by proton nuclear magnetic resonance (^1^H NMR) spectroscopy. The grafting amount of PBA was identified by comparing the integral areas of the characteristic proton signals of the G5 PAMAM dendrimer (2.2 to 3.4 ppm) and the aromatic protons of PBA (7.0 to 8.0 ppm), indicating that approximately 20 PBA molecules were grafted onto each G5 dendrimer (Fig. S1A). Likewise, the galactose substitution degree was calculated from the integration of galactose proton signals (3.4 to 4.5 ppm) relative to the methylene proton signals of the G3 PAMAM dendrimer (2.2 to 3.4 ppm), revealing that approximately one galactose molecule was conjugated to each G3 dendrimer (Fig. S1B). Furthermore, the ^1^H NMR spectrum of CSTD showed that approximately 10 G3-Gal molecules were assembled around each G5-PBA dendrimer (Fig. S2).

Next, 2-dimensional nuclear Overhauser effect spectroscopy was performed to verify the successful formation of the core–shell architecture of CSTD. As shown in Fig. 2A, distinct cross-peaks were observed between the aromatic protons of PBA and the galactose proton signals, indicating close spatial proximity between G5-PBA and G3-Gal. These results provide direct evidence for the successful assembly of the CSTD through dynamic boronate ester interactions, which is consistent with our previous findings [35]. Subsequently, atomic force microscopy was employed to analyze the height profile and surface morphology of the assembled CSTD. Atomic force microscopy images revealed well-dispersed spherical CSTD nanoparticles (Fig. 2B), and height profile analysis of representative nanoparticles revealed an average particle height of 11.8 nm (Fig. 2C), which is consistent with the expected dimensions of the CSTD. To further verify the chemical interactions responsible for CSTD assembly, Fourier transform infrared spectroscopy was recorded. Fourier transform infrared analysis revealed a boronic acid-related band at 1,357 cm^−1^ for G5-PBA and a galactose-associated peak at 970 cm^−1^ for G3-Gal (Fig. 2D). After assembly, a new absorption band appeared at 1,317 cm^−1^, which was related to boronate ester formation, further supporting the successful assembly of the CSTD.

**Fig. 2.**
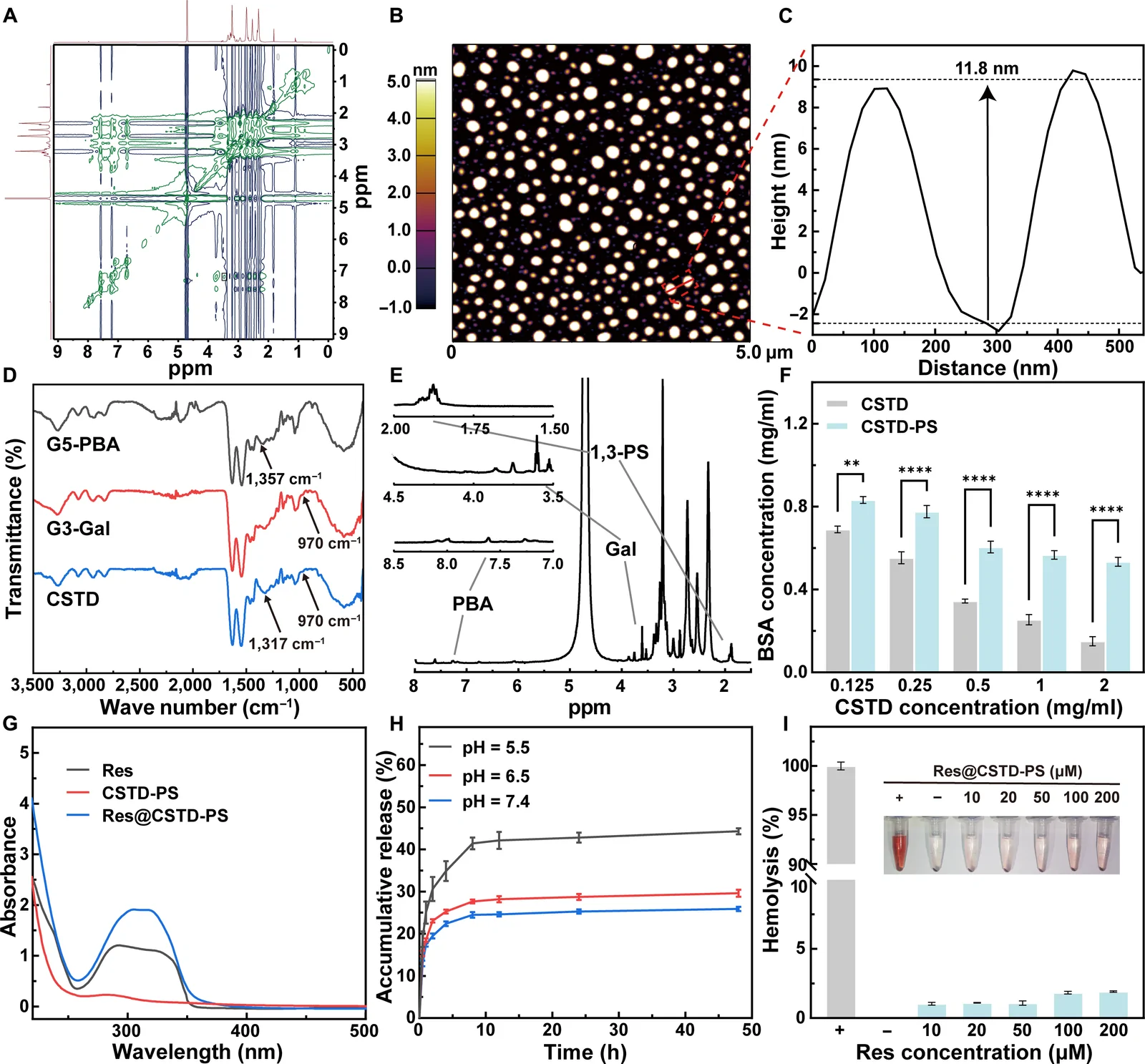
Construction and characterization of the responsive core–shell tecto dendrimer (CSTD)-based nanocarrier and Res@CSTD-PS nanocomplex. (A) Two-dimensional nuclear Overhauser effect spectroscopy (2D NOESY) spectrum of CSTD. (B) Atomic force microscopy (AFM) image and (C) corresponding height profile analysis of CSTD. (D) Fourier transform infrared (FTIR) spectra of phenylboronic acid-functionalized fifth-generation polyamidoamine (PAMAM) dendrimer (G5-PBA), galactose-modified third-generation PAMAM dendrimer (G3-Gal), and CSTD. (E) Proton nuclear magnetic resonance (^1^H NMR) spectrum of CSTD-PS. (F) Quantitative analysis of residual bovine serum albumin (BSA) in supernatants after incubation with CSTD and CSTD-PS (*n* = 3). (G) Ultraviolet–visible (UV–vis) spectra of CSTD-PS, free resveratrol (Res), and Res@CSTD-PS. (H) In vitro Res release from Res@CSTD-PS at pH = 7.4, 6.5, and 5.5 (*n* = 3). (I) Hemolysis assay of Res@CSTD-PS at different Res concentrations using red blood cells (RBCs) (*n* = 3). Inset: photographs of RBCs after treatment with Res@CSTD-PS at different Res concentrations followed by centrifugation.

Following successful construction of the CSTD, zwitterionic modification was carried out through conjugation with 1,3-PS onto the dendrimer surface to obtain CSTD-PS. Quantitative analysis of the ^1^H NMR spectra was performed by comparing the integral ratios between the characteristic proton signals of 1,3-PS (1.9 ppm) and PBA (7.0 to 8.0 ppm), revealing an average grafting number of approximately thirty-one 1,3-PS molecules per CSTD (Fig. 2E). Dynamic light scattering (DLS) measurements demonstrated that CSTD-PS possessed an average hydrodynamic diameter of 182.1 ± 1.06 nm and a polydispersity index of 0.443 ± 0.01 (Table S1). Compared with unmodified CSTD, CSTD-PS exhibited a slightly reduced hydrodynamic diameter, revealing that surface modification did not disrupt the integrity of the assembled nanostructure. In addition, only a marginal change in zeta potential was observed after 1,3-PS modification (29.0 ± 2.02 mV), suggesting that zwitterionic functionalization had little influence on the overall surface charge of the nanocarrier.

Despite the minimal change in zeta potential, the biological interfacial properties of CSTD-PS were expected to be markedly improved by the hydration layer formed by the surface zwitterionic groups. The coexistence of oppositely charged groups in the zwitterionic moieties can promote strong surface hydration, thereby reducing direct interactions between the nanocarrier surface and surrounding biomolecules. Such a hydrated interfacial layer is generally considered beneficial for reducing nonspecific protein adsorption and maintaining colloidal stability in biological environments. Therefore, the protein adsorption resistance of CSTD-PS was evaluated using bovine serum albumin (BSA) as a model protein, with unmodified CSTD serving as the control. As shown in Fig. 2F, CSTD-PS maintained a higher level of BSA in the supernatant than CSTD across the tested concentrations, suggesting improved resistance to nonspecific protein adsorption. However, compared with unmodified CSTD, CSTD-PS maintained significantly higher BSA concentrations in the supernatant at all tested concentrations (*P* < 0.01), revealing reduced nonspecific protein adsorption after zwitterionic modification. These results suggest that the introduction of 1,3-PS improved the resistance of CSTD-PS to BSA adsorption under the tested in vitro conditions.

### Fabrication and characterization of the Res@CSTD-PS nanocomplex

Based on the above-established CSTD-PS nanocarrier, Res was subsequently encapsulated through hydrophobic interactions to construct the Res@CSTD-PS nanocomplex. First of all, the incorporation of Res into CSTD-PS was measured by UV–vis spectroscopy (Fig. 2G). The Res@CSTD-PS nanocomplex exhibited a characteristic absorption peak of Res at 306 nm, confirming the presence of Res within the nanocarrier system. The Res LC and EE were determined as 8.4% and 82.1%, respectively (Table S2). Based on the quantitative analysis, each CSTD-PS nanocarrier was estimated to encapsulate 41 Res molecules, demonstrating the efficient drug-loading capability of the CSTD-PS nanocarrier. This drug-loading behavior is similar to that previously reported for CSTD-based platforms to accommodate small-molecule anticancer agents within their enlarged internal architecture [38,41]. These findings demonstrate the versatility of the CSTD architecture for the delivery of structurally distinct therapeutic agents. In addition, comparison with previously reported Res-based nanocarriers indicates that drug-loading characteristics vary substantially depending on carrier composition and formulation strategy [25,28]. Moreover, the physicochemical properties of Res@CSTD-PS were characterized by dynamic light scattering and zeta potential measurements (Table S1). Compared with CSTD-PS, Res@CSTD-PS exhibited an increased hydrodynamic diameter of 219.8 ± 5.30 nm with a reduced polydispersity index of 0.211 ± 0.05, suggesting the successful encapsulation of Res within the nanocarrier without compromising its colloidal stability. Meanwhile, the surface potential remained positive (29.1 ± 1.28 mV), which is favorable for maintaining nanoparticle stability and facilitating cellular internalization.

Given the dynamic boronate ester linkages within the CSTD structure, the pH-responsive release behavior in vitro of Res@CSTD-PS was subsequently investigated under physiological (pH = 7.4) and acidic conditions (pH = 6.5 and 5.5) (Fig. 2H). Res release exhibited a pH-dependent profile, with a markedly accelerated release under strongly acidic conditions. Approximately 44% of Res was released within 48 h at pH = 5.5, while moderate release was observed at pH = 6.5 (~29%), which was higher than that under physiological conditions (~25%). These results demonstrate the acid-responsive release capability of Res@CSTD-PS under the tested in vitro conditions, which mimic the acidic feature of the tumor microenvironment. Finally, the hemocompatibility of Res@CSTD-PS was evaluated. As shown in Fig. 2I, Res@CSTD-PS induced negligible hemolysis at all tested Res concentrations, with hemolysis ratios remaining below the accepted safety threshold of 5%. These results indicate that Res loading did not compromise the excellent blood compatibility of the nanocarrier, supporting its potential for systemic administration. Collectively, Res@CSTD-PS possesses efficient Res-loading capability, acid-responsive drug release behavior, and favorable biocompatibility, highlighting its potential as a promising nanoplatform for systemic HCC therapy.

### Res@CSTD-PS potentiates antitumor activity against HCC cells in vitro

To determine whether the CSTD-PS nanocarrier could potentiate the anticancer activity of Res, the in vitro antitumor activity of Res@CSTD-PS was investigated using mouse HCC Hepa1-6 cells. Cyanine 5 (Cy5)-labeled CSTD-PS (CSTD-PS-Cy5) was initially prepared as a fluorescent probe to monitor the intracellular delivery behavior of the nanocarrier. UV–vis spectroscopy revealed that Cy5-CSTD-PS exhibited the characteristic absorption peak of Cy5 at 650 nm, confirming the successful labeling of Cy5 onto the nanocarrier (Fig. S3). Meanwhile, quantitative analysis showed that the average Cy5 conjugation ratio was approximately 3.7 Cy5 molecules per CSTD-PS. After encapsulating Res, the cellular internalization behavior of Res@CSTD-PS-Cy5 was then evaluated in Hepa1-6 cells using confocal laser scanning microscopy. Obvious intracellular Cy5 fluorescence was observed in cells treated with Res@CSTD-PS-Cy5 (Fig. 3A). The fluorescence intensity gradually increased from 4 to 6 h and reached the maximum level at 6 h, followed by a decrease at 8 h. Quantitative analysis further confirmed the time-dependent cellular internalization profile of Res@CSTD-PS-Cy5 (Fig. S4). These results demonstrate that CSTD-PS can efficiently facilitate cellular uptake, providing a prerequisite for enhanced intracellular delivery of Res. In addition, we think that the intrinsic proton-buffering capacity of PAMAM dendrimers may also contribute to the intracellular delivery of Res. The protonatable tertiary amines within PAMAM dendrimers can buffer the acidic endosomal/lysosomal environment, potentially facilitating endosomal/lysosomal escape through the proton sponge effect [33]. Therefore, this intrinsic property of the PAMAM-based CSTD-PS may facilitate the intracellular release of encapsulated Res after cellular uptake. However, as lysosomal escape was not directly evaluated in the present study, its specific contribution to Res delivery remains to be further investigated.

**Fig. 3.**
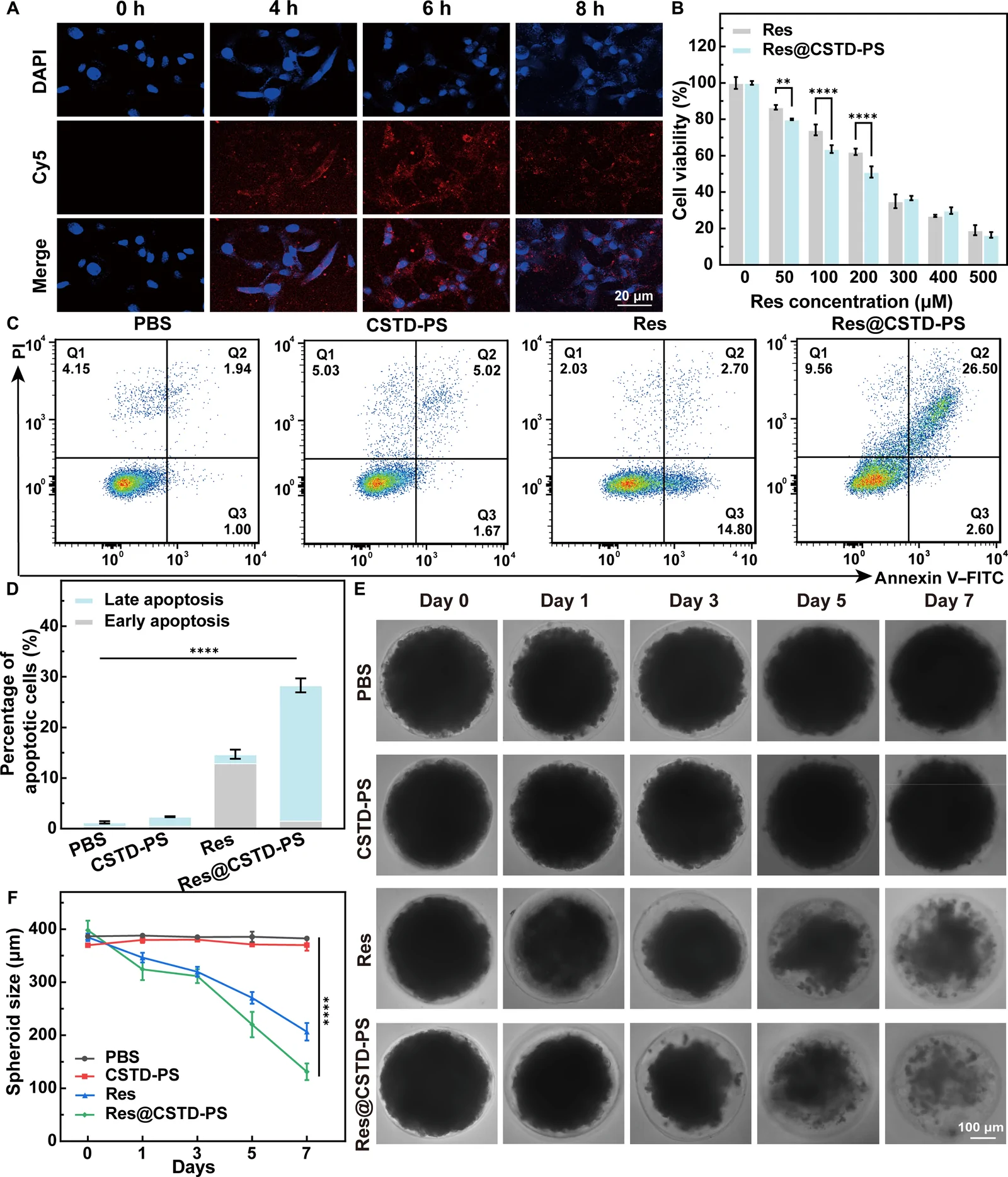
Evaluation of the intracellular delivery and antitumor activity of Res@CSTD-PS against hepatocellular carcinoma (HCC) cells in vitro. (A) Representative confocal images of Hepa1-6 cells treated with Res@CSTD-PS-Cy5 for 4, 6, and 8 h, respectively. Scale bar = 20 μm. (B) Cell viability of Hepa1-6 cells treated with free resveratrol (Res) or Res@CSTD-PS at different Res concentrations. (C) Representative flow cytometry plots and (D) quantitative analysis of apoptotic cell populations in Hepa1-6 cells treated with phosphate-buffered saline (PBS), CSTD-PS, free Res, or Res@CSTD-PS. (E) Representative images and (F) quantitative analysis of 3-dimensional (3D) Hepa1-6 tumor spheroids after treatment with PBS, CSTD-PS, free Res, or Res@CSTD-PS. Scale bar = 100 μm. Quantitative data are presented as mean ± SD (*n* = 3).

Subsequently, a Cell Counting Kit-8 assay was used to evaluate the in vitro cytotoxicity of Res@CSTD-PS. As expected, CSTD-PS alone exhibited negligible cytotoxicity even at high concentrations (Fig. S5), indicating the favorable biocompatibility of the nanocarrier. In contrast, both free Res and Res@CSTD-PS induced a concentration-dependent decrease in cell viability (Fig. 3B). Notably, within the tested concentration range ([Res]: 50 to 200 μM), Res@CSTD-PS displayed significantly enhanced cytotoxicity compared with free Res at equivalent Res concentrations (*P* < 0.01), resulting in a more pronounced reduction in cell viability. These results demonstrate that CSTD-PS-mediated delivery effectively potentiated the anticancer activity of Res against Hepa1-6 cells. To further elucidate whether the enhanced cytotoxicity of Res@CSTD-PS was associated with apoptosis induction, annexin V–fluorescein isothiocyanate/propidium iodide double staining was used to perform flow cytometric analysis. PBS or CSTD-PS groups exhibited minimal apoptotic and necrotic populations (Fig. 3C and D), suggesting the negligible cytotoxicity of the nanocarrier. In contrast, free Res induced an increased proportion of apoptotic cells, whereas Res@CSTD-PS treatment resulted in the highest percentage of apoptotic cell populations. Quantitative analysis further demonstrated that Res@CSTD-PS significantly enhanced Res-induced cell death compared with free Res (*P* < 0.0001). These findings indicate that CSTD-PS-mediated delivery potentiates the anticancer activity of Res by enhancing Res-induced apoptotic cell death in Hepa1-6 cells.

Next, a 3D Hepa1-6 tumor spheroid model was established to validate the antitumor efficacy of Res@CSTD-PS in a more physiologically relevant tumor model. During the treatment period, 3D spheroids treated with PBS or CSTD-PS maintained compact structures and exhibited minimal changes in size (Fig. 3E and F), indicating the negligible influence of the carrier itself on spheroid growth. By comparison, free Res induced progressive spheroid shrinkage and structural disruption, whereas Res@CSTD-PS resulted in the most pronounced inhibition of spheroid growth, with a significantly reduced spheroid volume compared with that with free Res (*P* < 0.0001). These results demonstrate that CSTD-PS-mediated delivery enhanced the therapeutic efficacy of Res in a 3D tumor model, further supporting the potential of Res@CSTD-PS for HCC treatment.

### Res@CSTD-PS induces coordinated metabolic stress and apoptotic responses in HCC cells

Metabolic reprogramming, particularly glycolytic enhancement, represents a key metabolic vulnerability in HCC, enabling tumor cells to sustain proliferation and adapt to unfavorable microenvironmental conditions [14,16]. Since Res exerted anticancer effects partly through metabolic regulation [42,43], we further investigated whether CSTD-PS-mediated delivery could enhance the metabolism-disrupting effects of Res in vitro. The glycolysis-associated protein HK2 was first examined. Reverse transcription quantitative polymerase chain reaction (RT-qPCR) (Fig. 4A) and western blot (WB) analyses (Fig. 4B and C) revealed that free Res reduced *Hk2* messenger RNA (mRNA) and HK2 protein expression compared with the PBS group and CSTD-PS group, whereas Res@CSTD-PS induced a more pronounced suppression of *Hk2* mRNA and HK2 protein expression. To determine whether these molecular changes were accompanied by the functional inhibition of glycolytic metabolism, extracellular lactate production was then quantified. As shown in Fig. S6, the extracellular lactate level remained comparable between the PBS and CSTD-PS groups, indicating that the nanocarrier itself had little effect on lactate production. In contrast, free Res treatment decreased extracellular lactate levels, while Res@CSTD-PS treatment resulted in a further reduction. Notably, the extracellular lactate level in the Res@CSTD-PS group was significantly lower than that in the PBS group (*P* < 0.0001). Together with the reduced HK2 expression, these findings provide functional evidence for the suppression of glycolytic activity by Res.

**Fig. 4.**
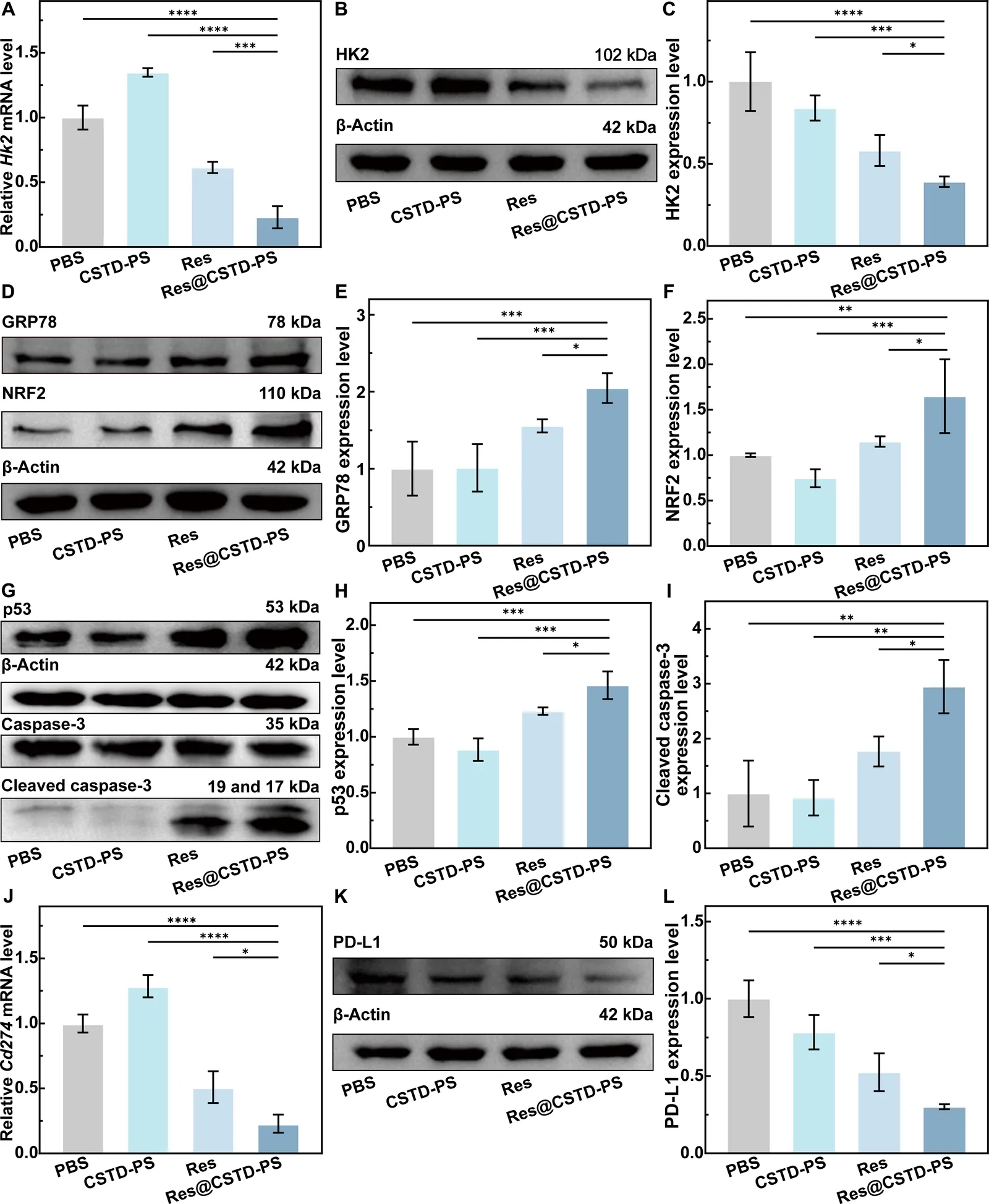
Res@CSTD-PS induces metabolic and stress-associated changes and apoptotic responses in hepatocellular carcinoma (HCC) cells. (A) Relative messenger RNA (mRNA) expression levels of *Hk2* in Hepa1-6 cells after various treatments. (B) Representative western blot bands and (C) quantitative analysis of hexokinase 2 (HK2) protein expression in Hepa1-6 cells after various treatments. (D) Representative western blot bands of glucose-regulated protein 78 (GRP78) and nuclear factor erythroid 2-related factor 2 (NRF2) in Hepa1-6 cells after different treatments. Corresponding quantitative analysis of (E) GRP78 and (F) NRF2 expression levels in Hepa1-6 cells after various treatments. (G) Representative western blot bands of p53, total caspase-3, and cleaved caspase-3 in Hepa1-6 cells after different treatments. Quantitative analysis of (H) p53 and (I) cleaved caspase-3 expression levels in Hepa1-6 cells after various treatments. (J) Relative *Cd274* mRNA expression level in Hepa1-6 cells after various treatments. (K) Representative western blot bands and (L) quantitative analysis of programmed death-ligand 1 (PD-L1) expression levels in Hepa1-6 cells after various treatments. Quantitative data are presented as mean ± SD (*n* = 3).

Given that metabolic perturbations are frequently associated with adaptive stress responses in tumor cells [16,44,45], we further examined whether Res@CSTD-PS-induced metabolic disruption was accompanied by changes in stress-associated proteins. As presented in Fig. 4D to F, both Res and Res@CSTD-PS treatment increased the expression of GRP78 and NRF2 compared with the PBS and CSTD-PS groups, with Res@CSTD-PS inducing the most pronounced up-regulation. The elevated expression of GRP78 and NRF2 suggested that Res@CSTD-PS treatment was associated with substantial intracellular stress and enhanced compensatory stress responses. However, persistent metabolic stress may exceed the adaptive capacity of HCC cells and further accompanied by alterations in apoptotic signaling pathways. Therefore, the activation of apoptosis-associated signaling pathways following Res@CSTD-PS treatment was subsequently investigated. RT-qPCR analysis revealed that Res@CSTD-PS treatment markedly increased *Trp53* mRNA expression compared with all other treatment groups (Fig. S7). WB analysis demonstrated that Res@CSTD-PS treatment resulted in the most pronounced up-regulation of p53 protein expression compared with all other groups (Fig. 4G and H). Furthermore, Res@CSTD-PS treatment obviously enhanced the cleavage of caspase-3 compared with free Res and other control groups (Fig. 4G and I), indicating the activation of the apoptotic execution pathway. Collectively, these data indicate that CSTD-PS-mediated delivery enhanced Res-associated metabolic perturbation and stress responses, which were accompanied by increased p53 expression, caspase-3 activation, and apoptotic cell death.

Metabolic reprogramming has been reported to regulate PD-L1 expression, while impaired glycolytic activity may attenuate tumor immune-evasive phenotypes [46–48]. Therefore, the effect of Res@CSTD-PS on tumor immune-evasive characteristics was further examined. As shown in Fig. 4J, *Cd274* mRNA levels were decreased following Res treatment, with the Res@CSTD-PS group exhibiting the strongest inhibitory effect compared with the control groups. Correspondingly, WB analysis revealed that Res treatment reduced PD-L1 expression compared with the PBS and CSTD-PS groups, whereas Res@CSTD-PS induced a more obvious down-regulation of PD-L1 (Fig. 4K and L). Taken together, these results suggest that CSTD-PS-mediated delivery not only enhanced the metabolic and apoptotic effects of Res but also reduced PD-L1 expression in HCC cells, suggesting a potential effect on immune-evasive characteristics.

### Res@CSTD-PS enhances tumor accumulation and therapeutic efficacy in vivo

Following the promising in vitro antitumor performance of Res@CSTD-PS, its therapeutic potential was further investigated in a Hepa1-6 xenograft tumor model. Res@CSTD-PS-Cy5 was initially used as a fluorescent probe to evaluate its in vivo delivery behavior and tumor accumulation capability. Following intravenous administration, the temporal distribution of free Cy5 and Res@CSTD-PS-Cy5 at tumor sites was monitored by whole-body fluorescence imaging (Fig. S8). Relative to free Cy5, Res@CSTD-PS-Cy5 produced markedly enhanced and sustained fluorescence signals at tumor sites, indicating improved tumor localization and prolonged intratumoral retention. This preferential tumor localization was further confirmed by ex vivo fluorescence imaging of excised tumors (Fig. S9), which showed stronger fluorescence signals in the Res@CSTD-PS-Cy5 group than in the free Cy5 group at both 0.5 and 48 h postinjection. Meanwhile, prominent fluorescence signals were seen in the liver and kidneys (Fig. S10), indicating the involvement of hepatic and renal pathways in the in vivo distribution and elimination of the nanocomplex. It should be noted that the Cy5 fluorescence signal reflects the biodistribution of the labeled nanocomplex rather than the direct tissue exposure of Res itself. Overall, these results demonstrate that CSTD-PS effectively enhanced tumor accumulation and prolonged intratumoral retention of the nanocomplex, providing a favorable foundation for improved therapeutic efficacy against HCC.

Subsequently, the therapeutic efficacy of Res@CSTD-PS was assessed in vivo. As illustrated in Fig. 5A, mice bearing subcutaneous Hepa1-6 tumors were randomly divided into 4 groups: PBS, CSTD-PS, free Res, or Res@CSTD-PS ([Res] = 5 mg/kg). The CSTD-PS group received an equivalent amount of carrier corresponding to the Res@CSTD-PS formulation. Tumor growth was monitored throughout the treatment period, and tumor tissues were collected at the end of treatment (day 16) for systematic evaluation of therapeutic efficacy and histological analysis. Compared with the PBS and CSTD-PS groups, free Res moderately inhibited tumor growth, whereas Res@CSTD-PS exhibited the strongest tumor growth inhibition effect, resulting in the smallest tumor volume on day 16 (Fig. 5B and Fig. S11). Consistently, excised tumors from the Res@CSTD-PS group showed significantly reduced weight compared with those from other groups (*P* < 0.05, Fig. 5C and D). Notably, no obvious body weight loss was observed in any group during the treatment period (Fig. S12), indicating the favorable systemic tolerance and biocompatibility of Res@CSTD-PS.

**Fig. 5.**
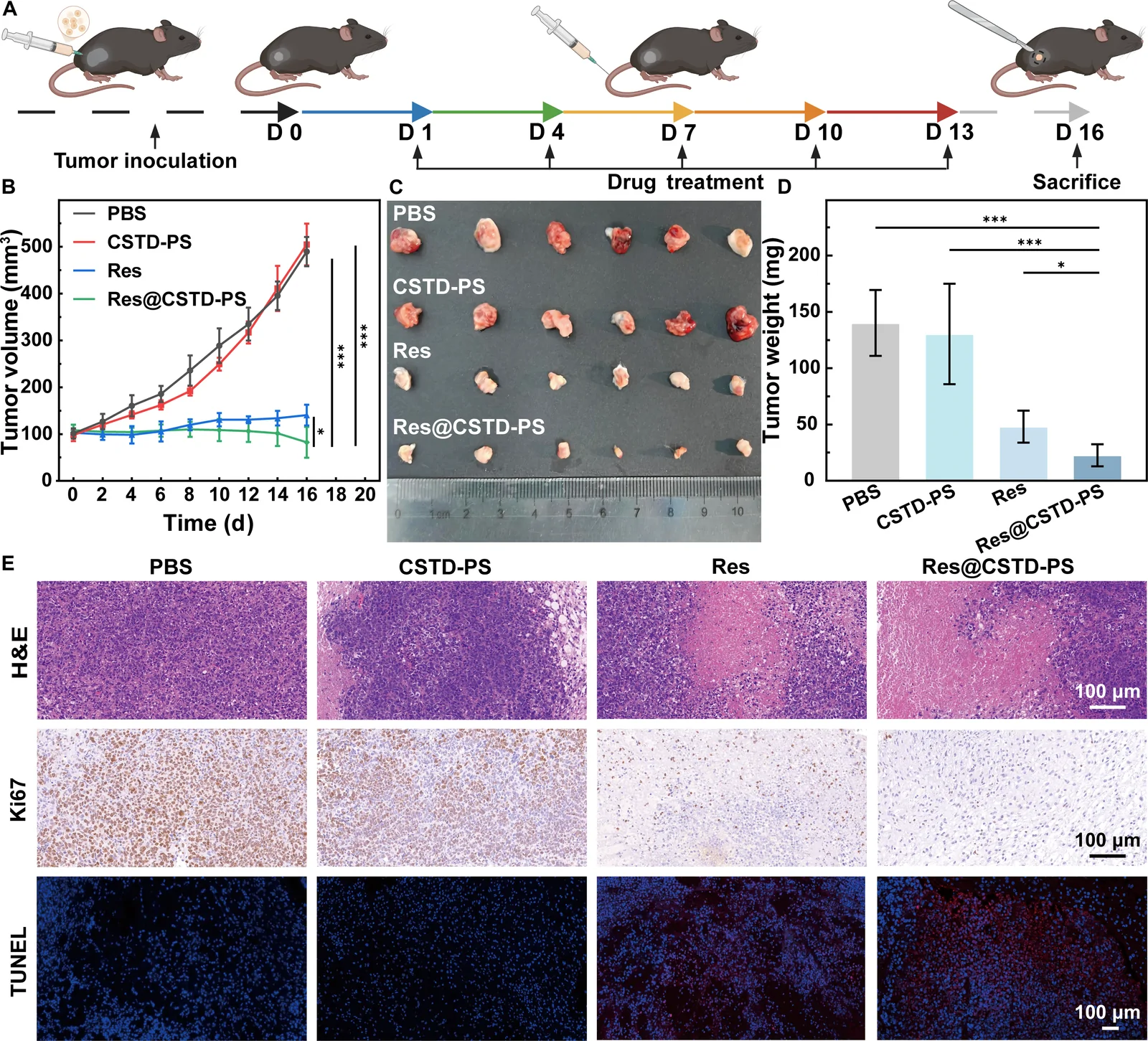
The therapeutic efficacy of Res@CSTD-PS against hepatocellular carcinoma (HCC) xenograft tumors. (A) Schematic illustration of the treatment protocol for Hepa1-6 tumor-bearing mice. (B) Tumor growth curves of mice after intravenous administration of phosphate-buffered saline (PBS), CSTD-PS, free resveratrol (Res), or Res@CSTD-PS (*n* = 6). (C) Photographs of excised tumors collected at day 16. (D) Tumor weights of different treatment groups at day 16 (*n* = 6). (E) Hematoxylin and eosin (H&E) staining, immunohistochemical Ki67 staining, and terminal deoxynucleotidyl transferase-mediated dUTP nick-end labeling (TUNEL) staining of tumor sections after various treatments. Scale bar = 100 μm.

To further confirm the therapeutic efficacy, tumor tissues were collected for histological analysis after treatment. H&E staining revealed extensive tumor cell damage, including reduced cellular density and increased necrotic regions, in the Res@CSTD-PS-treated group, whereas tumors from the PBS and CSTD-PS groups maintained intact cellular morphology (Fig. 5E). Free Res treatment induced moderate histological alterations, but to a lesser extent than Res@CSTD-PS. Coincidentally, immunohistochemical staining of Ki67 demonstrated markedly decreased proliferative activity in tumors treated with Res@CSTD-PS compared with free Res and control groups, indicating the strongest inhibition of tumor cell proliferation. Meanwhile, TUNEL staining displayed substantially increased apoptotic cells in the Res@CSTD-PS group, further confirming enhanced apoptosis induction in vivo and consistent with the enhanced apoptosis observed in vitro. The in vivo fluorescence imaging, tumor growth inhibition, and histological analyses demonstrate that Res@CSTD-PS achieves enhanced tumor accumulation and potent antitumor efficacy, supporting its potential for HCC therapy.

### Res@CSTD-PS induces coordinated metabolic and apoptotic responses and reduces PD-L1 expression in vivo

To further validate whether the metabolic-stress-associated mechanism observed in vitro could be reproduced in vivo, tumor tissues collected after treatment were subjected to immunohistochemical staining, immunofluorescence analysis, and WB examination. Consistent with the in vitro evaluation of glycolytic metabolism, the expression of glycolysis-related proteins in tumors was first examined. As shown in Fig. 6A, immunohistochemical staining revealed that GLUT1 expression was markedly reduced in tumors treated with Res@CSTD-PS compared with those in the PBS and CSTD-PS groups, while free Res induced a moderate suppression of GLUT1 expression, suggesting an enhanced glycolytic inhibitory effect after CSTD-PS-mediated delivery. Consistently, RT-qPCR (Fig. S13) and WB analysis (Fig. 6B) demonstrated that Res treatment reduced *Hk2* mRNA and HK2 protein expression, with Res@CSTD-PS exhibiting the strongest inhibitory effect among all groups. Similarly, immunohistochemical staining of PKM2 demonstrated a comparable expression pattern, with the most pronounced reduction observed in the Res@CSTD-PS-treated tumors compared with those in the PBS, CSTD-PS, and free Res groups (Fig. 6C). Taken together, these results indicate that CSTD-PS-mediated delivery reinforced the glycolytic inhibitory effect of Res in vivo, as evidenced by the suppression of key glycolysis-associated proteins and disruption of tumor metabolic homeostasis.

**Fig. 6.**
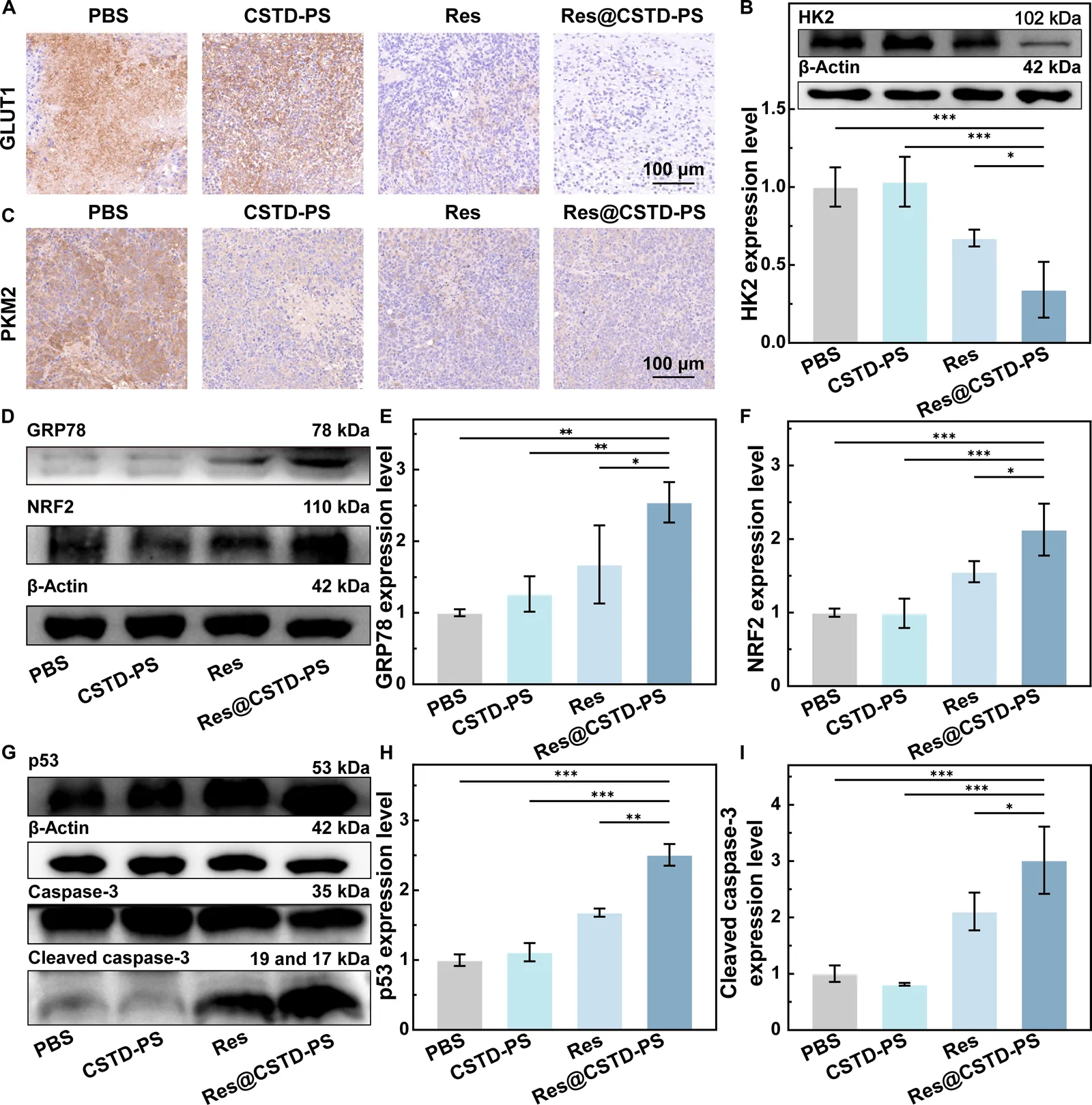
Metabolic and stress-associated changes and apoptotic responses in hepatocellular carcinoma (HCC) tumors following Res@CSTD-PS treatment. (A) Immunohistochemical staining of glucose transporter 1 (GLUT1) expression in tumor tissues after various treatments. Scale bar = 100 μm. (B) Western blot analysis of hexokinase 2 (HK2) expression in tumor tissues after various treatments. (C) Immunohistochemical staining of pyruvate kinase M2 (PKM2) expression in tumor tissues after various treatments. Scale bar = 100 μm. (D) Representative western blot bands of glucose-regulated protein 78 (GRP78) and nuclear factor erythroid 2-related factor 2 (NRF2) protein expression in tumor tissues after various treatments. Quantitative analysis of (E) GRP78 and (F) NRF2 expression levels in tumor tissues after various treatments. (G) Representative western blot bands of p53, total caspase-3, and cleaved caspase-3 in tumor tissues after various treatments. Quantitative analysis of (H) p53 and (I) cleaved caspase-3 expression levels in tumor tissues after various treatments. Quantitative data are presented as mean ± SD (*n* = 3).

In parallel with the observed suppression of glycolytic metabolism, stress-associated responses in tumor tissues were further investigated. WB analysis revealed that both Res and Res@CSTD-PS treatment increased the expression of GRP78 and NRF2 (Fig. 6D to F), with Res@CSTD-PS inducing the most pronounced up-regulation. These findings indicate that Res@CSTD-PS-induced metabolic perturbation was accompanied by a pronounced adaptive stress response within tumor tissues. Considering that persistent metabolic stress may eventually exceed cellular adaptive capacity and activate apoptotic signaling pathways [49,50], apoptosis-related proteins were subsequently examined. Res@CSTD-PS markedly increased both *Trp53* mRNA (*P* < 0.01) and p53 protein expression levels (*P* < 0.01) (Fig. S14 and Fig. 6G and H). Meanwhile, Res@CSTD-PS promoted caspase-3 cleavage (*P* < 0.05) compared with free Res and control groups (Fig. 6G and I), indicating enhanced activation of the apoptotic cascade in vivo.

In addition to metabolic and apoptotic regulation, the effect of Res@CSTD-PS on PD-L1 expression was assessed. Both RT-qPCR and WB analyses showed that Res treatment reduced *Cd274* mRNA and PD-L1 protein expression, whereas Res@CSTD-PS induced the most pronounced down-regulation of PD-L1 among all groups (Fig. S15). These results show that Res@CSTD-PS reduces PD-L1 expression in HCC tumors, which may suggest a potential modulation of immune-evasive characteristics. However, PD-L1 down-regulation alone does not establish enhanced antitumor immunity, and its functional immunological consequences require further validation through assessments of immune cell infiltration and T-cell activation. Taken together, these findings demonstrate that Res@CSTD-PS induces coordinated alterations in glycolytic metabolism, stress responses, apoptosis, and PD-L1 expression in vivo. However, it should be noted that the present in vivo study was conducted using a subcutaneous Hepa1-6 tumor model, which does not fully reproduce the liver-specific tumor microenvironment and immune cell composition of HCC. Further studies using orthotopic HCC models are warranted to validate the therapeutic effects and molecular responses of Res@CSTD-PS in a more physiologically relevant hepatic context.

### In vivo biosafety assessment of Res@CSTD-PS

The in vivo biosafety profile of Res@CSTD-PS was comprehensively assessed through hematological analysis and histopathological evaluation. Complete blood count analysis was performed to evaluate hematological parameters, including red blood cells, monocytes, lymphocytes, white blood cells, granulocytes, platelets, hemoglobin, hematocrit, and mean corpuscular volume (Fig. S16). No significant abnormalities were observed in hematological analysis after Res@CSTD-PS administration compared with the PBS control group, and all values remained within normal physiological ranges. Furthermore, H&E staining of major organs, including the liver, heart, spleen, kidneys, and lungs, revealed no obvious inflammatory lesions or pathological changes (Fig. S17). In short, these results demonstrate that Res@CSTD-PS exhibits favorable systemic biocompatibility and negligible in vivo toxicity at the administered dose, supporting its potential for biomedical application.

## Conclusion

In summary, we developed a pH-responsive zwitterion-modified CSTD nanocarrier (CSTD-PS) for the efficient delivery of Res to potentiate HCC therapy. The integration of dynamic boronate ester-mediated core–shell assembly and zwitterionic PS surface modification enabled efficient Res encapsulation, reduced nonspecific protein adsorption, improved biocompatibility, facilitated acid-responsive Res release, and enhanced tumor accumulation of the nanocomplex. Compared with free Res, Res@CSTD-PS exhibited enhanced cellular internalization, superior antitumor activity, and improved therapeutic efficacy. Mechanistically, Res@CSTD-PS disrupted tumor metabolic homeostasis by suppressing glycolysis-associated proteins, including HK2, GLUT1, and PKM2, while inducing adaptive stress responses characterized by elevated GRP78 and NRF2 expression. These metabolic and stress-associated changes were accompanied by increased p53 expression, caspase-3 activation, and apoptotic cell death. Moreover, Res@CSTD-PS reduced PD-L1 expression, suggesting potential attenuation of immune-evasive characteristics in HCC. Taken together, this work provides a promising nanomedicine strategy for improving the therapeutic efficacy of natural anticancer agents, offering new opportunities for HCC treatment.

## Data Availability

The data supporting the findings of this study are available from the corresponding authors upon reasonable request.

## References

[1] Huang DQ, Singal AG, Kanwal F, Lampertico P, Buti M, Sirlin CB, Nguyen MH, Loomba R. Hepatocellular carcinoma surveillance—Utilization, barriers and the impact of changing aetiology. Nat Rev Gastroenterol Hepatol. 2023;20(12):797–809. 10.1038/s41575-023-00818-8 37537332

[2] Singal AG, Kanwal F, Llovet JM. Global trends in hepatocellular carcinoma epidemiology: Implications for screening, prevention and therapy. Nat Rev Clin Oncol. 2023;20(12):864–884. 10.1038/s41571-023-00825-3 37884736

[3] Vogel A, Meyer T, Sapisochin G, Salem R, Saborowski A. Hepatocellular carcinoma. Lancet. 2022;400(10360):1345–1362. 10.1016/S0140-6736(22)01200-4 36084663

[4] Reig M, Forner A, Rimola J, Ferrer-Fàbrega J, Burrel M, Garcia-Criado Á, Kelley RK, Galle PR, Mazzaferro V, Salem R, et al. BCLC strategy for prognosis prediction and treatment recommendation: The 2022 update. J Hepatol. 2022;76(3):681–693. 10.1016/j.jhep.2021.11.018 PMC8866082 34801630

[5] Finn RS, Qin S, Ikeda M, Galle PR, Ducreux M, Kim T-Y, Kudo M, Breder V, Merle P, Kaseb AO, et al. Atezolizumab plus bevacizumab in unresectable hepatocellular carcinoma. N Engl J Med. 2020;382(20):1894–1905. 10.1056/NEJMoa1915745 32402160

[6] Yang C, Zhang H, Zhang L, Zhu AX, Bernards R, Qin W, Wang C. Evolving therapeutic landscape of advanced hepatocellular carcinoma. Nat Rev Gastroenterol Hepatol. 2023;20(4):203–222. 10.1038/s41575-022-00704-9 36369487

[7] Kalasekar SM, VanSant-Webb CH, Evason KJ. Intratumor heterogeneity in hepatocellular carcinoma: Challenges and opportunities. Cancer. 2021;13(21):5524. 10.3390/cancers13215524 PMC8582820 34771685

[8] Llovet JM, Castet F, Heikenwalder M, Maini MK, Mazzaferro V, Pinato DJ, Pikarsky E, Zhu AX, Finn RS. Immunotherapies for hepatocellular carcinoma. Nat Rev Clin Oncol. 2021;19(3):151–172. 10.1038/s41571-021-00573-2 34764464

[9] Park S, Hall MN. Metabolic reprogramming in hepatocellular carcinoma: Mechanisms and therapeutic implications. Exp Mol Med. 2025;57(3):515–523. 10.1038/s12276-025-01415-2 PMC11958682 40025169

[10] Llovet JM, Kelley RK, Villanueva A, Singal AG, Pikarsky E, Roayaie S, Lencioni R, Koike K, Zucman-Rossi J, Finn RS. Hepatocellular carcinoma. Nat Rev Dis Primers. 2021;7(1):6. 10.1038/s41572-020-00240-3 PMC13537433 33479224

[11] Liberti MV, Locasale JW. The Warburg effect: How does it benefit cancer cells? Trends Biochem Sci. 2016;41(3):211–218. 10.1016/j.tibs.2015.12.001 PMC4783224 26778478

[12] DeBerardinis RJ, Chandel NS. Fundamentals of cancer metabolism. Sci Adv. 2016;2(5): Article e1600200. 10.1126/sciadv.1600200 PMC4928883 27386546

[13] Hanahan D. Hallmarks of cancer: New dimensions. Cancer Discov. 2022;12(1):31–46. 10.1158/2159-8290.CD-21-1059 35022204

[14] Feng J, Li J, Wu L, Yu Q, Ji J, Wu J, Dai W, Guo C. Emerging roles and the regulation of aerobic glycolysis in hepatocellular carcinoma. J Hepatol. 2020;39(1):126. 10.1186/s13046-020-01629-4 PMC7336654 32631382

[15] Li J, Huang Y, Li J, Shi M, Xiao Y, Du F, Hu G. Metabolic reprogramming-driven resistance to multi-kinase inhibitors in hepatocellular carcinoma: Molecular mechanisms and therapeutic opportunities. Mol Cancer. 2026;25(1):37. 10.1186/s12943-026-02578-w PMC12918548 41593671

[16] Faubert B, Solmonson A, DeBerardinis RJ. Metabolic reprogramming and cancer progression. Science. 2020;368(6487):eaaw5473. 10.1126/science.aaw5473 PMC7227780 32273439

[17] Levy JMM, Towers CG, Thorburn A. Targeting autophagy in cancer. Nat Rev Cancer. 2017;17(9):528–542. 10.1038/nrc.2017.53 PMC5975367 28751651

[18] Ren B, Kwah MX-Y, Liu C, Ma Z, Shanmugam MK, Ding L, Xiang X, Ho PC-L, Wang L, Ong PS, et al. Resveratrol for cancer therapy: Challenges and future perspectives. Cancer Lett. 2021;515:63–72. 10.1016/j.canlet.2021.05.001 34052324

[19] Ko J-H, Sethi G, Um J-Y, Shanmugam MK, Arfuso F, Kumar AP, Bishayee A, Ahn KS. The role of resveratrol in cancer therapy. Int J Mol Sci. 2017;18(12):2589. 10.3390/ijms18122589 PMC5751192 29194365

[20] Wu S-X, Xiong R-G, Huang S-Y, Zhou D-D, Saimaiti A, Zhao C-N, Shang A, Zhang Y-J, Gan R-Y, Li H-B. Effects and mechanisms of resveratrol for prevention and management of cancers: An updated review. Crit Rev Food Sci Nutr. 2022;63(33):12422–12440. 10.1080/10408398.2022.2101428 35852215

[21] Jang JY, Im E, Kim ND. Mechanism of resveratrol-induced programmed cell death and new drug discovery against cancer: A review. Int J Mol Sci. 2022;23(22):13689. 10.3390/ijms232213689 PMC9697740 36430164

[22] Bao R, Wang T, Jiang W. Resveratrol for cancer treatment: Effects on metabolism and immune cells. Biomolecules. 2026;16(1):118. 10.3390/biom16010118 PMC12839258 41594658

[23] Lee Y, Lee S-H, Choi D, Nam H-S, Kim KD, Choi MH, Cho M-K, Lee Y-J. Resveratrol targets glycolytic enzymes HK II and PKM2 to promote concurrent apoptotic and necrotic cell death in malignant melanoma. Curr Issues Mol Biol. 2025;47(12):1006. 10.3390/cimb47121006 PMC12732220 41614770

[24] Berman AY, Motechin RA, Wiesenfeld MY, Holz MK. The therapeutic potential of resveratrol: A review of clinical trials. NPJ Precis Oncol. 2017;1:35. 10.1038/s41698-017-0038-6 PMC5630227 28989978

[25] Boroumand R, Haghighizadeh A, Salmasi Z, Rajabi O. Cyclodextrin-mediated delivery of resveratrol: Recent advances, therapeutic potential, and future perspectives. Carbohydr Polym. 2026;388: Article 125526. 10.1016/j.carbpol.2026.125526 42493097

[26] Mitchell MJ, Billingsley MM, Haley RM, Wechsler ME, Peppas NA, Langer R. Engineering precision nanoparticles for drug delivery. Nat Rev Drug Discov. 2020;20(2):101–124. 10.1038/s41573-020-0090-8 PMC7717100 33277608

[27] Wilhelm S, Tavares AJ, Dai Q, Ohta S, Audet J, Dvorak HF, Chan WCW. Analysis of nanoparticle delivery to tumours. Nat Rev Mater. 2016;1(5):16014.

[28] Lv Y, Li W, Liao W, Jiang H, Liu Y, Cao J, Lu W, Feng Y. Nano-drug delivery systems based on natural products. Int J Nanomedicine. 2024;19:541–569. 10.2147/IJN.S443692 PMC10802180 38260243

[29] Wang J, Li D, Fan Y, Shi M, Yang Y, Wang L, Peng Y, Shen M, Shi X. Core–shell tecto dendrimers formed *via* host–guest supramolecular assembly as pH-responsive intelligent carriers for enhanced anticancer drug delivery. Nanoscale. 2019;11(46):22343–22350. 10.1039/c9nr08309j 31728477

[30] Uppuluri S, Swanson DR, Piehler LT, Li J, Hagnauer GL, Tomalia DA. Core–shell tecto(dendrimers): I. Synthesis and characterization of saturated shell models. Adv Mater. 2000;12(11):796–800.

[31] Tomalia DA. Birth of a new macromolecular architecture: Dendrimers as quantized building blocks for nanoscale synthetic polymer chemistry. Prog Polym Sci. 2005;30(3–4):294–324.

[32] Mekuria SL, Song C, Ouyang Z, Shen M, Janaszewska A, Klajnert-Maculewicz B, Shi X. Synthesis and shaping of core–shell tecto dendrimers for biomedical applications. Bioconjug Chem. 2021;32(2):225–233. 10.1021/acs.bioconjchem.1c00005 33459011

[33] Mintzer MA, Grinstaff MW. Biomedical applications of dendrimers: A tutorial. Chem Soc Rev. 2011;40(1):173–190. 10.1039/b901839p 20877875

[34] Song C, Shen M, Rodrigues J, Mignani S, Majoral J-P, Shi X. Superstructured poly(amidoamine) dendrimer-based nanoconstructs as platforms for cancer nanomedicine: A concise review. Coord Chem Rev. 2020;421:213463.

[35] Liu J, Wang X, Li X, Ni C, Liu L, Bányai I, Shi X, Song C. Structural and property characterizations of dual-responsive core–shell tecto dendrimers for tumor penetration and gene delivery applications. Macromol Rapid Commun. 2024;45(17):2400251. 10.1002/marc.202400251 38813898

[36] Chen F, Kong L, Wang L, Fan Y, Shen M, Shi X. Construction of core–shell tecto dendrimers based on supramolecular host–guest assembly for enhanced gene delivery. J Mater Chem B. 2017;5(43):8459–8466. 10.1039/c7tb02585h 32264513

[37] Song C, Xiao Y, Ouyang Z, Shen M, Shi X. Efficient co-delivery of microRNA 21 inhibitor and doxorubicin to cancer cells using core–shell tecto dendrimers formed *via* supramolecular host–guest assembly. J Mater Chem B. 2020;8(14):2768–2774. 10.1039/d0tb00346h 32154812

[38] Song C, Zhan M, Ouyang Z, Yao Y, Gao Y, Shen M, Shi X. Core-shell tecto dendrimer-mediated cooperative chemoimmunotherapy of breast cancer. J Control Release. 2023;358:601–611. 10.1016/j.jconrel.2023.05.021 37201799

[39] Song C, Ouyang Z, Guo H, Qu J, Gao Y, Xia J, Shen M, Shi X. Core–shell tecto dendrimers enable enhanced tumor MR imaging through an amplified EPR effect. Biomacromolecules. 2021;22(5):2181–2188. 10.1021/acs.biomac.1c00262 33848141

[40] Song C, Ouyang Z, Gao Y, Guo H, Wang S, Wang D, Xia J, Shen M, Shi X. Modular design of multifunctional core-shell tecto dendrimers complexed with copper(II) for MR imaging-guided chemodynamic therapy of orthotopic glioma. Nano Today. 2021;41:101325.

[41] Gong J, Song C, Li G, Guo Y, Wang Z, Guo H, Xia J, Tao Y, Shi Q, Shi X, et al. Ultrasound-enhanced theranostics of orthotopic breast cancer through a multifunctional core–shell tecto dendrimer-based nanomedicine platform. Biomater Sci. 2023;11(12):4385–4396. 10.1039/d3bm00375b 37158209

[42] Li S, Yin S, Ding H, Shao Y, Zhou S, Pu W, Han L, Wang T, Yu H. Polyphenols as potential metabolism mechanisms regulators in liver protection and liver cancer prevention. Cell Prolif. 2022;56(1): Article e13346. 10.1111/cpr.13346 PMC9816945 36229407

[43] Brockmueller A, Sameri S, Liskova A, Zhai K, Varghese E, Samuel SM, Büsselberg D, Kubatka P, Shakibaei M. Resveratrol’s anti-cancer effects through the modulation of tumor glucose metabolism. Cancer. 2021;13(2):188. 10.3390/cancers13020188 PMC7825813 33430318

[44] Hetz C, Papa FR. The unfolded protein response and cell fate control. Mol Cell. 2018;69(2):169–181. 10.1016/j.molcel.2017.06.017 29107536

[45] Hayes JD, Dinkova-Kostova AT, Tew KD. Oxidative stress in cancer. Cancer Cell. 2020;38(2):167–197. 10.1016/j.ccell.2020.06.001 PMC7439808 32649885

[46] Chang C-H, Qiu J, O’Sullivan D, Buck MD, Noguchi T, Curtis JD, Chen Q, Gindin M, Gubin MM, van der Windt GJW, et al. Metabolic competition in the tumor microenvironment is a driver of cancer progression. Cell. 2015;162(6):1229–1241. 10.1016/j.cell.2015.08.016 PMC4864363 26321679

[47] Li X, Wenes M, Romero P, Huang SC-C, Fendt S-M, Ho P-C. Navigating metabolic pathways to enhance antitumour immunity and immunotherapy. Nat Rev Clin Oncol. 2019;16(7):425–441. 10.1038/s41571-019-0203-7 30914826

[48] Jiang Z, Liu Z, Li M, Chen C, Wang X. Increased glycolysis correlates with elevated immune activity in tumor immune microenvironment. EBioMedicine. 2019;42:431–442. 10.1016/j.ebiom.2019.03.068 PMC6491961 30935888

[49] Dalseno D, Gajic N, Flanagan L, Tait SWG. Cell death and cancer: Metabolic interconnections. Cell Rep. 2025;44(6): Article 115804. 10.1016/j.celrep.2025.115804 PMC12187904 40489330

[50] Zhang W, Shi Y, Oyang L, Cui S, Li S, Li J, Liu L, Li Y, Peng M, Tan S, et al. Endoplasmic reticulum stress—A key guardian in cancer. Cell Death Discov. 2024;10(1):343. 10.1038/s41420-024-02110-3 PMC11289465 39080273

