# Supplementary material for: Resveratrol-Loaded Responsive Core–Shell Tecto Dendrimers Potentiate Hepatocellular Carcinoma Therapy
Source: Biomater Res. 2026 Oct 14;30:0428. doi: 10.34133/bmr.0428 (PMC13662274; doi:10.34133/bmr.0428)
Supplement: Supplementary file 1 [file bmr.0428.f1.docx]

Supporting Information

**Resveratrol-loaded responsive core-shell tecto dendrimers potentiate hepatocellular carcinoma therapy**

*Wenchao Liu,* ^1^*^+^ Junjie Liu,*^2+^ *Zhenze Zheng,^1^ Wenqing Man,^4^ Xiaoyang Zhang,^4^ Xiaoyi Zheng, ^4^ Yupeng Wang,^3^ Yao Shi,^3^* Qinghai Lin,^1^* Cong Song*^1, 4^***

^1^ Department of Clinical Laboratory, Shandong Cancer Hospital and Institute, Shandong First Medical University & Shandong Academy of Medical Sciences, Jinan 250117, China.

^2^ DWI-Leibniz-Institute for Interactive Materials, Institute for Technical and Macromolecular Chemistry, RWTH Aachen University, Aachen 52074, Germany.

^3^ Biomedical Sciences College & Shandong Medical Biotechnology Research Center, National Health Commission Key Laboratory of Biotechnology Drugs, Shandong First Medical University & Shandong Academy of Medical Sciences, Jinan 250117, China.

^4^ Medical Science and Technology Innovation Center, Shandong First Medical University & Shandong Academy of Medical Sciences, Jinan 250117, China.

**^+^** These authors contributed equally to this work.

* Corresponding author.

E-mail addresses: [shiyao@sdfmu.edu.cn](mailto:shiyao@sdfmu.edu.cn) (Y. Shi), [linqh@sd-cancer.com](mailto:linqh@sd-cancer.com) (Q. Lin) and [songcong@sdfmu.edu.cn](mailto:songcong@sdfmu.edu.cn) (C. Song)

**Supplementary Materials and Methods**

**Materials**

Generation 5 (G5) poly(amidoamine) (PAMAM) dendrimers and Generation 3 (G3) poly(amidoamine) (PAMAM) dendrimers were from Weihai Chenyuan Molecular New Material Co., LtD (Weihai, China). D-Galacturonic acid (Gal) and Cy5-NHS were purchased from Shanghai Yuanye Biotechnology Co., Ltd. (Shanghai, China). 1-(3 Dimethylaminopropyl)-3-ethylcarbodiimide hydrochloride (EDC·HCl), N- hydroxysuccinimide (NHS) were supplied by Shanghai Aladdin Biochemical Technology Co., Ltd. (Shanghai, China). 1,3-Propane sultone (PS), Resveratrol (Res), and 4-(bromomethyl)phenylboronic acid (PBA) were supplied by Shanghai Macklin Biochemical Co., Ltd. Dimethyl sulfoxide (DMSO) was provided by Sinopharm Chemical Reagent Co., Ltd. (Shanghai, China). Regenerated cellulose dialysis membranes with molecular weight cut-offs (MWCOs) of 1000 Da or 10000 Da were acquired from Fisher Scientific (Pittsburgh, PA). Hepa1-6 cells were obtained from the liver cancer cell line of C57/L mice and provided by National Collection of Authenticated Cell Cultures. Fetal bovine serum (FBS) was purchased from SeraPure (Bavaria, Germany). Dulbecco’s Modified Eagle Medium (DMEM), 0.25% trypsin-EDTA, penicillin-streptomycin, Cell Cycle Detection Kit, and Annexin V-FITC/PI Apoptosis Detection Kit were obtained from Jiangsu Keygen Biotech Co., Ltd. (Nanjing, China). DAPI was bought from Abcam Trading Co., Ltd (Shanghai, China). The Cell Counting Kit-8 (CCK-8) was acquired from Shanghai Hongye Biotechnology Co., Ltd. (Shanghai, China). Antibodies against HK2, GRP78, and NRF2 were bought from Proteintech Group, Inc. (Wuhan, China). The PD-L1 antibody was bought from ABclonal Biotechnology Co., Ltd. (Wuhan, China). Antibodies against p53 and Caspase-3 were purchased from Cell Signaling Technology (Danvers, USA). All primers used for RT-qPCR were synthesized by Genewiz Biotechnology Co., Ltd. (Suzhou, China).

**Preparation of G3-Gal, G5-PBA, and Responsive Core-Shell Tecto Dendrimers (CSTDs)**

The G3-Gal, G5-PBA, and CSTDs were prepared following procedures described in our earlier reports [35]. G3-Gal was synthesized by coupling Gal to G3 PAMAM dendrimers through EDC/NHS-mediated amidation, followed by dialysis (MWCO = 1000 Da) and lyophilization. G5-PBA was prepared by conjugating PBA to G5 PAMAM dendrimers in DMSO at 60 °C, followed by dialysis (MWCO = 1000 Da) and lyophilization. CSTDs were then assembled by mixing aqueous solutions of G5-PBA and G3-Gal through dynamic boronate ester formation, purified by dialysis (MWCO = 10,000 Da), lyophilized, and stored at -20 °C until further use.

**Preparation of CSTD-PS-Cy5 and Res@CSTD-PS-Cy5**

First of all, the CSTD-PS with surface amine groups were reacted with 10 molar equivalents of Cyanine 5 (Cy5) in anhydrous dimethyl sulfoxide (DMSO) under vigorous magnetic stirring for 24 h. And then, each reaction mixture was dialyzed against water (2 L/per time, total 9 times) for 3 days using a dialysis membrane with a MWCO of 1000 Da. After freeze-drying, the CSTD-PS-Cy5 were obtained and stored at -20 ℃ for further use. Subsequently, Res@CSTD-PS-Cy5 was prepared using the same procedure as described for Res@CSTD-PS.

**General Characterization Techniques**

^1^H nuclear magnetic resonance (^1^H NMR) spectra and two-dimensional nuclear Overhauser effect spectroscopy (2D NOESY) measurements were recorded on a Bruker AV 800 NMR spectrometer (Karlsruhe, Germany). All samples were dissolved in D_2_O before measurements. Fourier transform infrared (FTIR) spectra were recorded on a Nicolet 6700 FTIR spectrophotometer (Thermo Electron Corporation, Madison, WI). Samples were mixed with milled KBr crystals and pressed to form 13-mm diameter disks before measurements. Zeta potential and dynamic light scattering (DLS) measurements were performed using a Malvern Zetasizer Nano ZS model ZEN3600 (Worcestershire, UK) coupled with a standard 633-nm laser. All samples were dispersed in water before measurements. Atomic force microscopy (AFM) images were obtained using an Asylum Research AFM system (Asylum Research, Santa Barbara, CA, USA). Samples were prepared by depositing diluted aqueous suspensions onto silicon wafers and drying under a nitrogen atmosphere before measurements. UV-vis spectra were collected using a Lambda 25 UV-vis spectrophotometer (Perkin Elmer, Waltham, MA). All samples were dispersed in water before measurements.

**Protein Adsorption Resistance Assay**

The protein adsorption resistance of CSTD-PS was evaluated using bovine serum albumin (BSA) as a model protein. Briefly, CSTD-PS or CSTD solutions at different concentrations (0.125, 0.25, 0.5, 1, and 2 mg/mL, 500 μL) were mixed with 500 μL of BSA solution (2 mg/mL) and incubated at 37 °C for 4 h. After incubation, the mixtures were centrifuged at 8000 rpm for 15 min, and the supernatants were collected. The remaining BSA concentration was quantified using a BCA protein assay kit according to the manufacturer’s instructions, and absorbance was measured using a microplate reader.

**Hemolysis Assay**

Blood was collected from healthy C57 mice *via* orbital sinus into anticoagulant tubes. Red blood cells (RBCs) were pelleted by centrifugation (3000 rpm, 5 min) and washed several times with saline until the supernatant became clear. After removing the supernatant, the RBCs were resuspended in 5 mL of PBS. In 1.5 mL centrifuge tubes, 100 μL of the RBC suspension was mixed with 900 μL of ultrapure water (positive control, pc), PBS (negative control, nc), or Res@CSTD-PS solutions at various Res concentrations (10, 20, 50, 100, 200 μM). The mixtures were incubated at 37 °C for 2 h, then centrifuged at 13,000 rpm for 15 min. The absorbance of the supernatant at 540 nm was measured. The hemolysis rate was calculated as: Hemolysis (%) = (Ds – Dnc) / (Dpc – Dnc) × 100%.

**Cell Culture**

Hepa1-6 cells were routinely cultured and passaged in DMEM medium supplemented with 10% FBS, 1% penicillin-streptomycin, and 1% sodium pyruvate (named DMEM^++^), at 37 °C in a 5% CO_2_.

**Cellular Uptake Assay**

Hepa1-6 cells were seeded in confocal dishes at 8 × 10^4^ cells per well in 500 μL DMEM^++^ and incubated overnight. The next day, the medium was replaced with fresh DMEM++ containing the Res@CSTD-PS-Cy5 ([Res] = 100 μM) and incubated for 0, 4, 6, or 8 h. After incubation, cells were washed three times with PBS, fixed with 1 mL of tissue fixative per well, washed again, and stained with DAPI. Fluorescence images were acquired using a confocal laser scanning microscopy (CLSM, ZEISS Celldiscoverer 7), and the fluorescence intensity was quantified using ImageJ (1.8.0.345).

**Cytotoxicity Assay**

The cytotoxicity of Res@CSTD-PS, Res, and CSTD-PS was evaluated using the Cell Counting Kit-8 (CCK-8) assay. Briefly, Hepa1-6 cells were seeded in 96-well plates at a density of 5,000 cells per well in 100 μL of DMEM^++^ and incubated overnight. The next day, the medium was replaced with fresh DMEM^++^ containing different Res concentrations ([Res] = 0, 50, 100, 200, 300, 400, and 500 μM). The corresponding amounts of CSTD-PS were applied according to the Res concentration in Res@CSTD-PS. After 24 h, the medium was replaced with CCK-8 working solution (10 μL CCK-8 in 100 μL DMEM^++^) and incubated for another 2 h. Absorbance at 450 nm was recorded using a microplate reader (Thermo Scientific Multiskan FC), with untreated Hepa1-6 cells as the control.

**Cell apoptosis Assay**

Hepa1-6 cells were seeded in 6-well plates at 2 × 10^5^ cells per well and incubated overnight. The next day, the medium was replaced with DMEM^++^ containing different treatments (PBS, CSTD-PS, free Res, Res@CSTD-PS) and incubated for 24 h. The Res concentration was maintained at 100 μM in both free Res and Res@CSTD-PS groups, and the CSTD-PS group received an equivalent amount of carrier corresponding to that in the Res@CSTD-PS formulation. Subsequently, the cells in each well were washed, collected, resuspended in 500 μL of binding buffer, and added with 5 μL of Annexin V-FITC and 5 μL of PI according to the kit instruction. Each sample was incubated for 15 min in the dark and measured by Thermo Fisher Scientific Attune NxT acoustic focusing flow cytometer (Waltham, MA). Cells treated with PBS were used as control. For each sample, 2 × 10^4^ cells were counted, and each measurement was repeated for 3 times.

**Reverse Transcription Quantitative Polymerase Chain Reaction (RT-qPCR) Analysis**

Hepa1-6 cells were seeded in 6-well plates at 2 × 10^5^ cells per well and incubated overnight. The next day, the medium was replaced with DMEM^++^ containing different treatments (PBS, CSTD-PS, free Res, Res@CSTD-PS) and incubated for 24 h. The Res concentration was maintained at 100 μM in both free Res and Res@CSTD-PS groups, and the CSTD-PS group received an equivalent amount of carrier corresponding to that in the Res@CSTD-PS formulation. Cells were then harvested (300 × g, 5 min), and total RNA was extracted and reverse-transcribed according to the manufacturer’s protocol. The obtained cDNA, SYBR Green qPCR mix, and gene-specific primers were mixed in a 20 μL reaction system, and qPCR was performed on a real-time PCR instrument (Tianlong GENTIER 96) using the pre-programmed protocol.

**Western Blot Analysis**

Hepa1-6 cells were seeded in 6-well plates at 2 × 10^5^ cells per well and incubated overnight. The next day, the medium was replaced with DMEM^++^ containing different treatments (PBS, CSTD-PS, free Res, Res@CSTD-PS) and incubated for 24 h. The Res concentration was maintained at 100 μM in both free Res and Res@CSTD-PS groups, and the CSTD-PS group received an equivalent amount of carrier corresponding to that in the Res@CSTD-PS formulation. Cells were harvested (300 × g, 5 min), washed twice with PBS, and lysed with RIPA buffer on ice for 30 min. After centrifugation, the supernatant was collected, and protein concentration was determined using a BCA protein assay kit. Equal amounts of protein samples were mixed with SDS-PAGE loading buffer, denatured at 95 °C for 8 min, and subjected to western blot analysis according to standard protocols. The target proteins were detected using specific primary antibodies and corresponding secondary antibodies, with β-actin used as the loading control.

***In Vitro* Assessment of Extracellular Lactate Production**

Hepa1-6 cells were seeded in six-well plates at a density of 1 × 10^5^ cells per well in 2 mL of DMEM^++^ and cultured overnight. The next day, the medium was replaced with DMEM^++^ containing different treatments (PBS, CSTD-PS, free Res, Res@CSTD-PS) and incubated for 24 h. The Res concentration was maintained at 100 μM in both free Res and Res@CSTD-PS groups, and the CSTD-PS group received an equivalent amount of carrier corresponding to that in the Res@CSTD-PS formulation. Lactate content in the culture supernatants was measured using a commercial assay kit according to the manufacturer’s instructions.

***In Vivo* Biodistribution Study**

The male C57BL/6 mice (~8 weeks old) were provided by Beijing Vital River Laboratory Animal Technology Co., Ltd. (Beijing, China). One week later, mice (≈ 24 g per mouse) were utilized to establish the Hepa1-6 subcutaneous tumor model. In brief, 3 × 10^6^ Hepa1-6 cells resuspended in 100 μL of PBS were injected into the right hind leg of each mouse. When tumors reached approximately 100 mm^3^, the mice were used for *in vivo* fluorescence imaging studies. Firstly, free Cy5 and Res@CSTD-PS-Cy5 with identical Cy5 concentrations ([Cy5] = 0.8 mg/kg) were subjected to fluorescence imaging to confirm comparable fluorescence intensity. Tumor-bearing mice with similar tumor volumes were randomly divided into two groups (free Cy5 and Res@CSTD-PS-Cy5). Background fluorescence images were recorded before injection. Subsequently, 100 μL of free Cy5 or Res@CSTD-PS-Cy5 was intravenously administered *via* the tail vein. The injected formulations contained an equivalent Cy5 dose of 0.8 mg/kg, corresponding to a Res dose of 5 mg/kg in the Res@CSTD-PS-Cy5 group. Whole-body fluorescence images were collected at predetermined time points (0.5, 1, 2, 4, 6, 12, 24, and 48 h post-injection). Tumor-associated fluorescence intensity was quantitatively analyzed after normalization to the pre-injection background signal. In addition, a separate biodistribution experiment was also performed. At the selected time point (0.5 h and 48 h), mice were euthanized, and major organs (heart, liver, spleen, lungs, and kidneys) as well as tumors were collected for *ex vivo* fluorescence imaging.

***In Vivo* Biosafety Investigation**

The healthy male C57BL/6 mice were randomly divided into four groups (PBS, CSTD-PS, free Res, and Res@CSTD-PS) and used for *in vivo* biosafety evaluation. One week later, each mouse received a 100 μL intravenous injection of the corresponding formulation *via* the tail vein. The Res dose was set at 5 mg/kg, and the corresponding amounts of CSTD-PS in the Res@CSTD-PS formulation were used as the carrier dose. At 48 h post-injection, mice were euthanized, and whole blood samples were collected for hematological analysis. Major organs, including the heart, liver, spleen, lungs, and kidneys, were harvested, fixed in tissue fixative, and subjected to histological examination by hematoxylin and eosin (H&E) staining.

**Table S1**. Hydrodynamic sizes and zeta potentials of CSTD-based nanocarrier and nanocomplex.

| Samples | Hydrodynamic size (nm) | Polydispersity index (PDI) | Zeta potential (mV) |
| --- | --- | --- | --- |
| CSTD | 220.4 ± 1.23 | 0.27 ± 0.12 | 30.1 ± 0.76 |
| CSTD-PS | 182.1 ± 1.06 | 0.443 ± 0.01 | 29.0 ± 2.02 |
| Res@CSTD-PS | 219.8 ± 5.30 | 0.211 ± 0.05 | 29.1 ± 1.28 |

*Data are provided as mean ± SD (n = 3).

**Table S2**. The loading content (LC) and encapsulation efficiency (EE) of Res@CSTD-PS.

| Sample | LC (%) | EE (%) |
| --- | --- | --- |
| Res@CSTD-PS | 8.4 | 82.1 |

**Figure S1**. ^1^H NMR spectra of (A) G5-PBA and (B) G3-Gal.

**Figure S2**. ^1^H NMR spectrum of CSTDs.

**Figure S3**. UV-vis absorption spectra of CSTD-PS and CSTD-PS-Cy5.

**Figure S4.** Quantification of intracellular Cy5 fluorescence intensity in Hepa1-6 cells treated with Res@CSTD-PS-Cy5 for 4, 6, and 8 h (n = 3).

**Figure S5.** Cell viability of Hepa1-6 cells treated with CSTD-PS alone at concentrations equivalent to those of Res used in the cytotoxicity assay (n = 6).

**Figure S6.** Extracellular L-lactate (L-LA) concentrations in Hepa1-6 cell culture supernatants after different treatments.

**Figure S7.** Relative mRNA expression levels of *Trp53* in Hepa1-6 cells after various treatments (n = 3).

**Figure S8.** (A) Representative whole-body fluorescence images and of mice after intravenous injection of free Cy5 or Res@CSTD-PS-Cy5 at different time points (0, 0.5, 1, 2, 4, 6, 12, 24, and 48 h). (B) Quantitative analysis of fluorescence intensity at tumor sites over time.

**Figure S9.** (A) Representative fluorescence images of excised tumors and (B) quantitative analysis of tumor-associated fluorescence intensity at 0.5 and 48 h post-injection (n = 3).

**Figure S10.** *Ex vivo* fluorescence imaging of major organs after intravenous administration of free Cy5 or Res@CSTD-PS-Cy5 at 0.5 and 48 h.

**Figure S11.** Individual tumor growth curves of Hepa1-6 tumor-bearing mice after different treatments (n = 6).

**Figure S12.** Body weight changes of Hepa1-6 tumor-bearing mice during the treatment period (n = 6).

**Figure S13.** Relative mRNA expression levels of *Hk2* in tumor issues after various treatments (n = 3).

**Figure S14.** Relative mRNA expression levels of *Trp53* in tumor issues after various treatments (n = 3).

**Figure S15.** (A) Relative *Cd274* mRNA expression levels in tumor issues after various treatments. (B) Representative western blot bands and (C) quantitative analysis of PD-L1 protein expression in tumor issues after various treatments. (n = 3)

**Figure S16.** The blood routines analysis of healthy mice in different treatment groups including levels of (A) WBC, (B) RBC, (C) HGB, (D) PLT, (E) Mon, (F) LYM, (G) Gran, (H) HCT, and (I) MCV. (n = 3). Orange areas indicate the referred normal ranges for healthy mice obtained from Ruiyu, Inc. (Shanghai, China) using Hematology Analyzer (Mindray, BC-2800 vet).

**Figure S17.** H&E-stained slices of major organs (heart, liver, spleen, lung, and kidney) of healthy mice at 48 h post different treatments (scale bar = 100 μm).
